# Supplementary material for: A novel sample handling system for dissolution dynamic nuclear polarization experiments
Source: Magn Reson (Gott). 2021 Jun 4;2(1):387–94. doi: 10.5194/mr-2-387-2021 (PMC10539747; doi:10.5194/mr-2-387-2021)
Supplement: The supplement related to this article is available online at: https://doi.org/10.5194/mr-2-387-2021-supplement. [file mr-2-387-supplement.pdf]

Supplement of Magn. Reson., 2, 387–394, 2021
https://doi.org/10.5194/mr-2-387-2021-supplement
© Author(s) 2021. CC BY 4.0 License.

Open Access
MAGNETIC RESONANCE

Supplement of

A novel sample handling system for dissolution dynamic nuclear polarization experiments

Thomas Kress et al.

Correspondence to: Dennis Kurzbach (dennis.kurzbach@univie.ac.at)

The copyright of individual parts of the supplement might differ from the article licence.

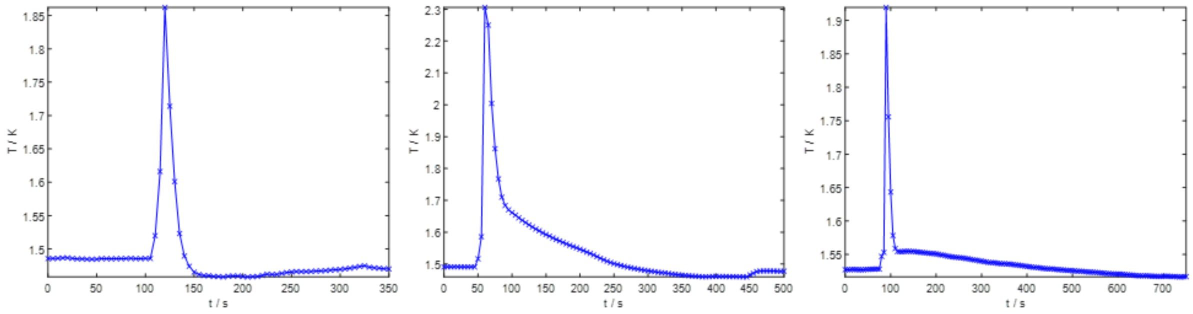

Figure S1. Temperature profiles for three different dissolutions. Note how the major temperature shock decays within a minute. Depending on the liquid helium level within the VTI, the system needs between one and 10 minutes to fully equilibrate after the dissolution experiment.

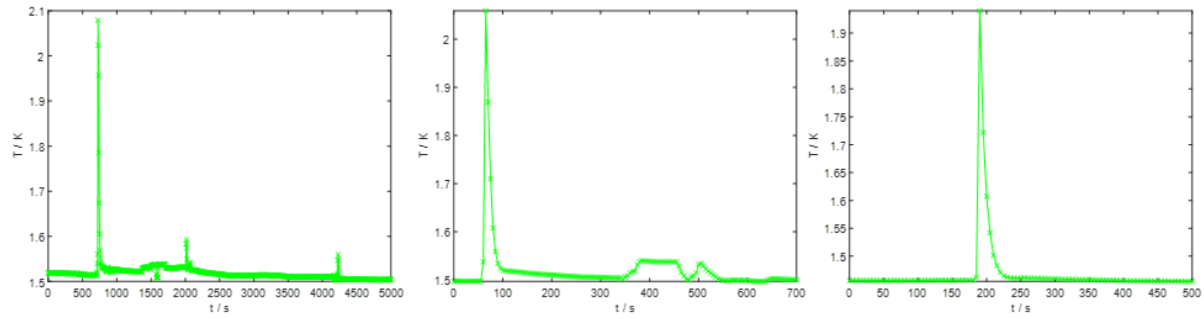

Figure S2. Temperature profiles for three different sample insertions. Note how the major temperature shock decays within a minute. As for dissolutions, depending on the liquid helium level within the VTI, the system needs between one and 10 minutes to fully equilibrate after the insertion.

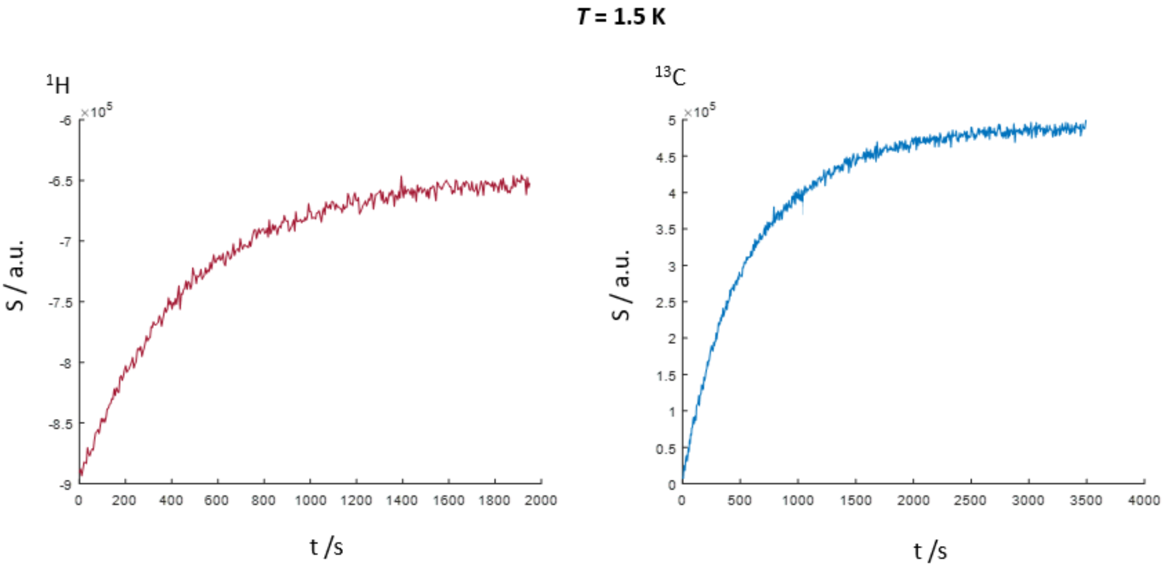

Figure S3. DNP build-up curves for ^{1}H and ^{13}C nuclei recorded at 1.5 K, at a TEMPOL concentration of 40 mM and a 50 μL sample (10% H_2O, 40% D_2O, 50% glycerol-d8, 3 M sodium acetate).

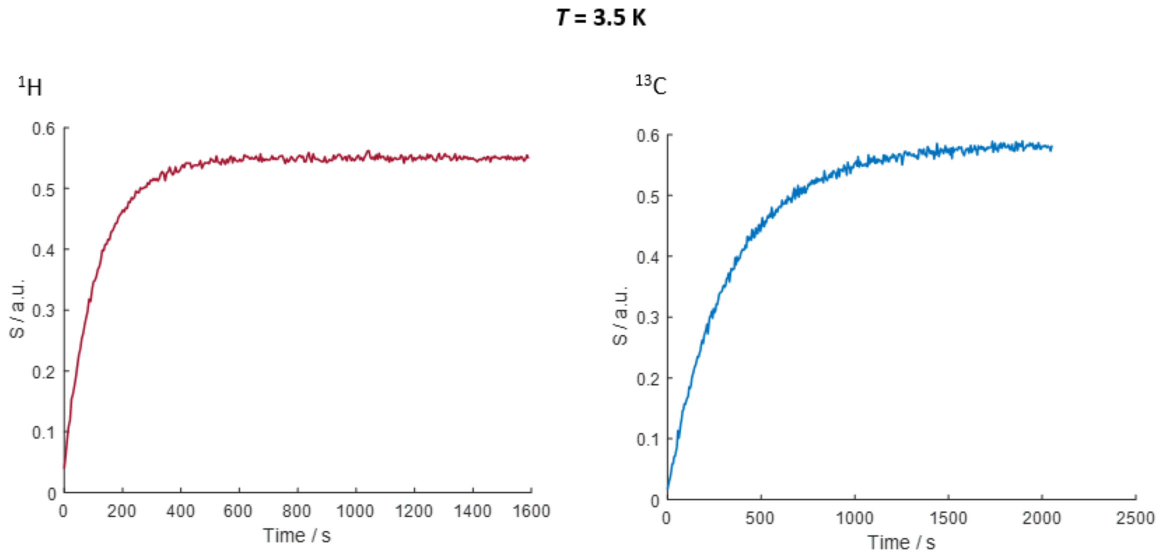

Figure S4. DNP build-up curves for ^{1}H and ^{13}C nuclei recorded at 3.5 K, at a TEMPOL concentration of 40 mM and a 50 μL sample (10% H_2O, 40% D_2O, 50% glycerol-d8, 3 M sodium acetate).

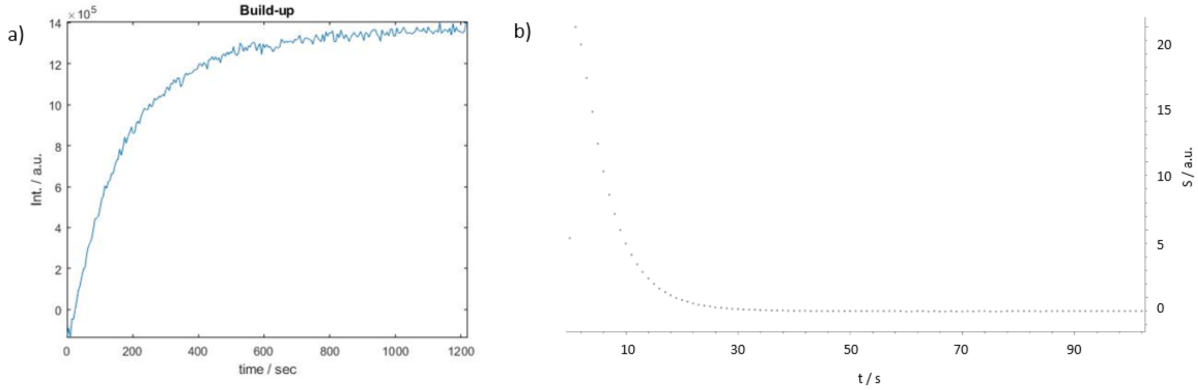

Figure S5. a) DNP build-up curves for ^{1}H nuclei recorded at 1.5 K, at a TEMPOL concentration of 40 mM and a 150 μL sample (10% H_2O, 40% D_2O, 50% glycerol-d8). b) Signal decay of the hyperpolarized water line upon injection into a 500 MHz Bruker NMR spectrometer. The signal enhancement was >800-fold in comparison to thermal equilibrium.

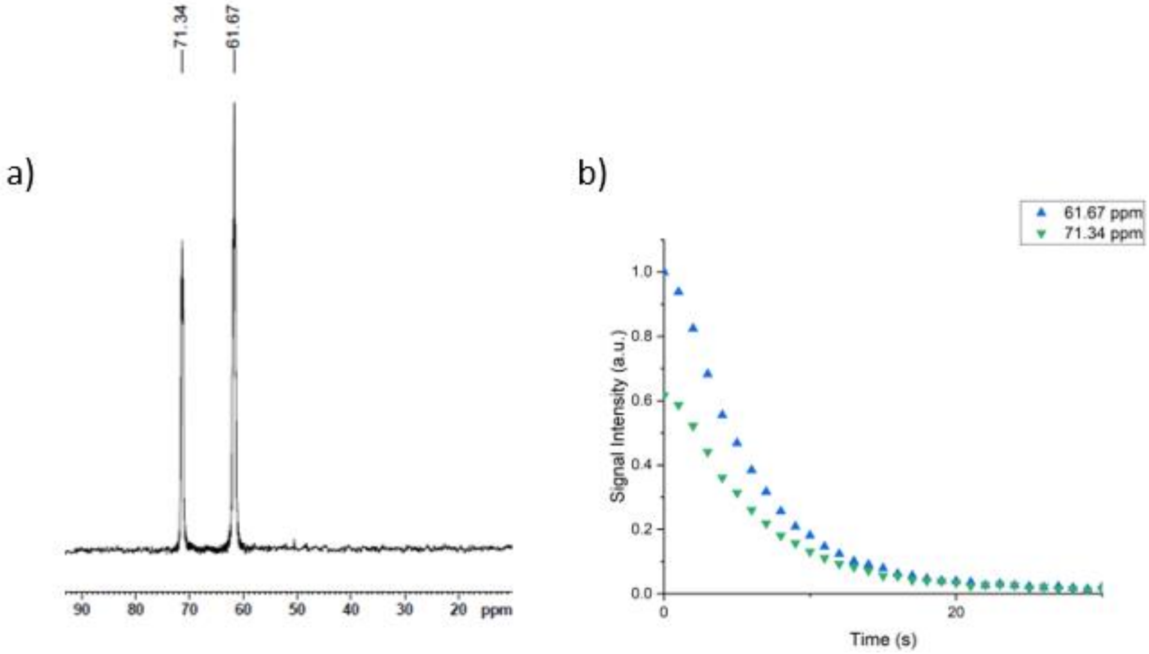

Figure S6. a) ^{13}C-hyperpolarized NMR signal of glycerol-d8 upon injection into a 500 MHz Bruker NMR spectrometer. b) Signal decay of the two glycerol signals. The signal enhancement was >3000 in comparison to the signal in thermal equilibrium.

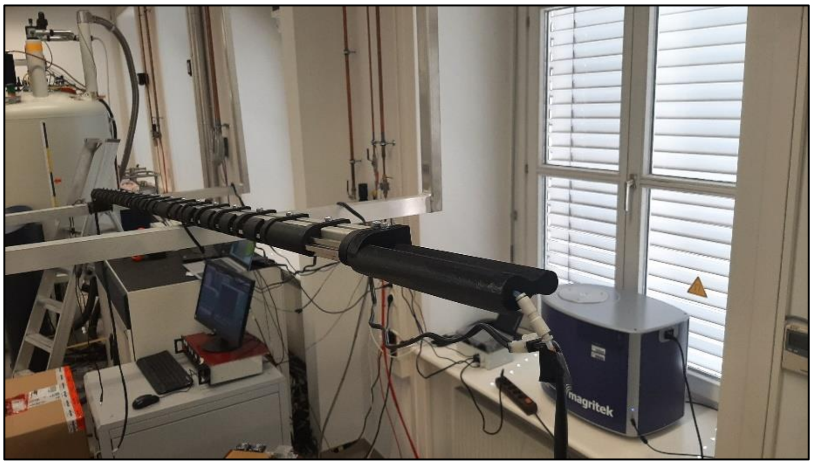

Figure S7. Picture of the magnetic tunnel used to transfer the sample to the 500 MHz NMR spectrometer. (Images by M. Negroni.)

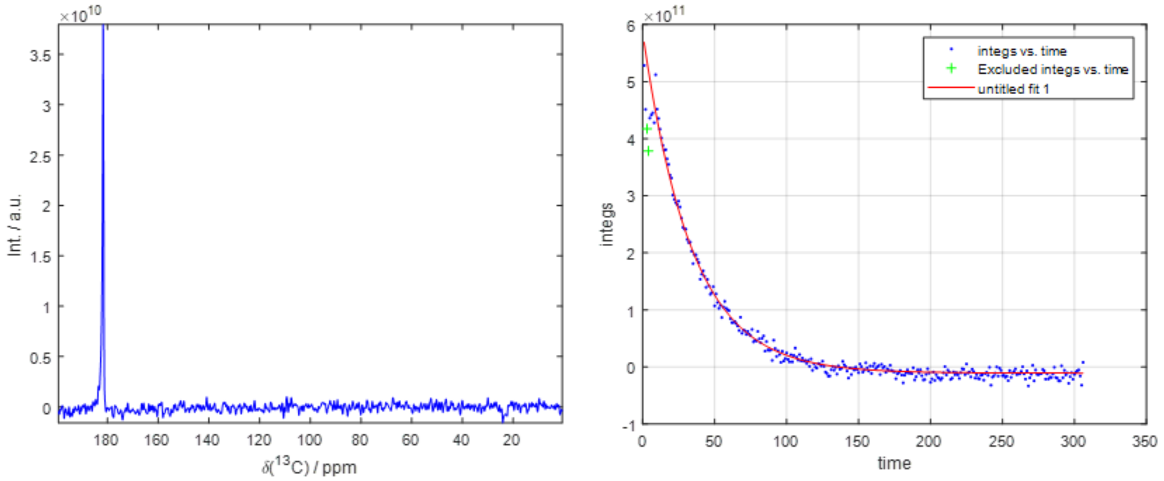

Figure S8. a) ^{13}C-hypoerpolarized NMR signal of sodium acetate upon injection into a 500 MHz Bruker NMR spectrometer. b) Signal decay of the two acetate signal.

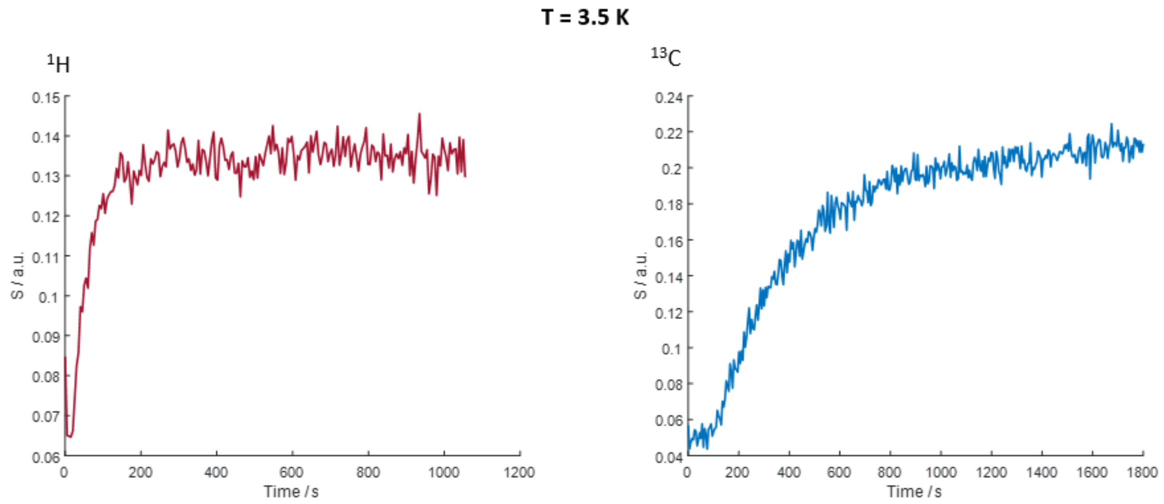

Figure S9. DNP build-up curves for ^{1}H and ^{13}C nuclei recorded at 3.5 K, at a TEMPOL concentration of 70 mM and a 50 µL sample (10% H_2O, 40% D_2O, 50% glycerol-d8, 3 M sodium acetate).

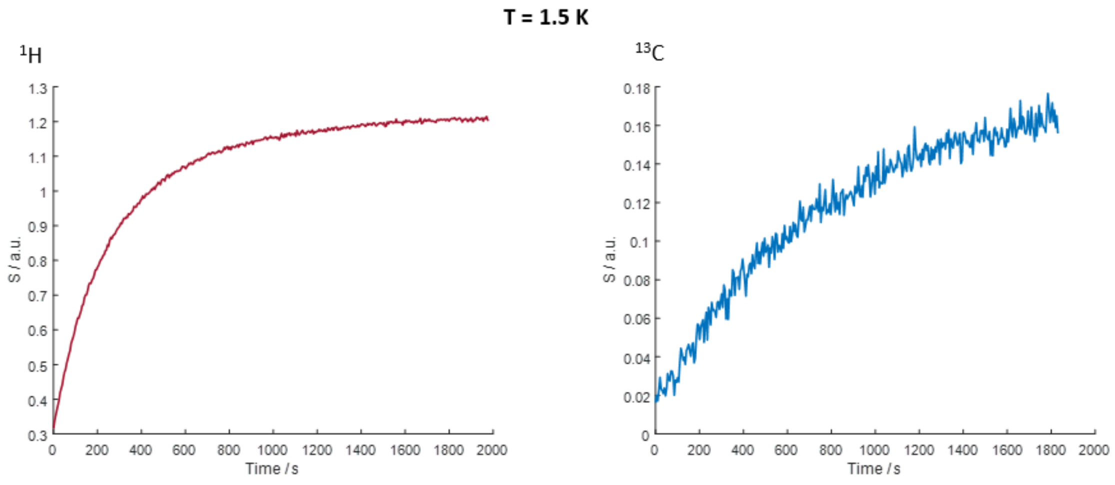

Figure S10. DNP build-up curves for ^{1}H and ^{13}C nuclei recorded at 1.5 K, at a TEMPOL concentration of 70 mM and a 50 μL sample (10% H_2O, 40% D_2O, 50% glycerol-d8, 3 M sodium acetate).
